# Attenuated neural activity in processing decision‐making feedback in uncertain conditions in patients with mild cognitive impairment

**DOI:** 10.1002/alz.093395

**Published:** 2025-01-09

**Authors:** Mang Zhang, Ying Zhang, luchun wang, Yaonan Zheng, Huizi Li, Yuhan Xie, Xiaozhen Lv, Xin Yu, Huali Wang

**Affiliations:** ^1^ Peking University Institute of Mental Health (Sixth Hospital), Beijing China; ^2^ Peking University, Beijing, Beijing China; ^3^ Beijing Anding Hospital, Beijing, Beijing China; ^4^ Peking University, Beijing China; ^5^ National Clinical Research Center for Mental Disorders, Beijing China

## Abstract

**Background:**

Individuals with mild cognitive impairment (MCI) have decreased cognitive function, which makes them prone to making inappropriate decisions in complex and uncertain situations. However, there is currently no study being undertaken to investigate the potential neural mechanisms for processing decision‐making feedback in MCI patients. The present study aimed to explore the potential neural correlates during feedback evaluation during decision‐making under risk and ambiguity in MCI.

**Method:**

Nineteen individuals with MCI and twenty age‐matched HCs were enrolled. Decision‐making performance under risk and ambiguity was examined with the modified game of dice task (GDT) and an Iowa gambling task (IGT). Using task‐related EEG data, reward positivity (RewP) and feedback P3 (fb‐P3) were used to characterize participants’ motivation and allocation of cognitive resources. Also, response time and event‐related oscillation (ERO) was used to evaluate information processing speed, and the potent of post‐feedback information integration and behavioral modulation.

**Result:**

MCI patients had lower RewP (p = 0.022) and fb‐P3 (p = 0.045) amplitudes in the GDT than HCs. Moreover, the amount and valence of feedback modulated the RewP (p = 0.008; p = 0.017) and fb‐P3 (p < 0.001; p < 0.001). In the IGT, in addition to the significantly reduced fb‐P3 observed in MCI patients (p = 0.010), the amount and valence of feedback modulated the RewP (p = 0.002; p = 0.020). Furthermore, MCI patients took longer to make decisions (t = 2.15, p = 0.041). The ERO analysis revealed that delta power was reduced in MCI (GDT: p = 0.045; p = 0.011).

**Conclusion:**

The findings suggest that, during feedback evaluation when making risky and ambiguous decisions, motivation, allocation of cognitive resources, information processing and neuronal excitability were attenuated in MCI. It implies that neural activity related to decision making was compromised in MCI.

